# Ultrasonographic Assessment of the Diaphragm and the Effects of Smoking on Respiratory Function in Individuals Attending a Smoking Cessation Center

**DOI:** 10.3390/jcm15051950

**Published:** 2026-03-04

**Authors:** Ahmet Utus, Semiramis Ozyilmaz, Turgay Karatas, Nurullah Dag, Gurkan Ural, Ipek Balikci Cicek, Murat Kılıc

**Affiliations:** 1 Department of Physiotherapy and Rehabilitation, Faculty of Health Sciences, Inonu University, 44280 Malatya, Turkey; ahmet.utus@inonu.edu.tr; 2 Department of Cardiopulmonary Physiotherapy and Rehabilitation, Institute of Health Sciences, Bezmialem Vakif University, 34093 Istanbul, Turkey; 3 Department of Physiotherapy and Rehabilitation, Faculty of Health Sciences, Bezmialem Vakif University, 34093 Istanbul, Turkey; sozyilmaz@bezmialem.edu.tr; 4 Department of Anatomy, Faculty of Medicine, Inonu University, 44000 Malatya, Turkey; 5 Department of Radiology, Faculty of Medicine, Inonu University, 44069 Malatya, Turkey; nurullah.dag@inonu.edu.tr (N.D.); gurkan.ural@inonu.edu.tr (G.U.); 6 Biostatistics Department, Faculty of Medicine, Inonu University, 44000 Malatya, Turkey; ipek.balikci@inonu.edu.tr; 7 Department of Thoracic Surgery, Faculty of Medicine, Inonu University, 44000 Malatya, Turkey; murat.kilic@inonu.edu.tr

**Keywords:** smoking, diaphragm muscle, ultrasonography, pulmonary function, six-minute walk test

## Abstract

**Background**: Smoking adversely affects pulmonary function and systemic health; however, its impact on diaphragm muscle morphology and its relationship with functional capacity and psychosocial outcomes in individuals without clinically diagnosed respiratory disease remain unclear. This study aimed to evaluate diaphragm muscle thickness in smokers and to investigate its associations with pulmonary function, functional capacity, sleep quality, and depression. **Methods:** This cross-sectional observational study included 20 smokers and 20 age-matched never-smokers. Pulmonary function was assessed using spirometry. Functional capacity was evaluated with the 6-Minute Walk Test (6 MWT) and the 30 s sit-to-stand test (30 s STST). Sleep quality and depression were assessed using the Pittsburgh Sleep Quality Index (PSQI) and the Beck Depression Inventory (BDI). Inspiratory and expiratory diaphragm muscle thicknesses were measured by ultrasonography. Between-group comparisons and correlation analyses were performed. Results: Smokers exhibited significant impairments in all assessed parameters except expiratory diaphragm thickness compared with controls (*p* < 0.05). Large to very large effect sizes were observed for FEV_1_, FEF_25–75_%, functional capacity, and inspiratory diaphragm thickness. Inspiratory diaphragm thickness showed moderate to strong positive correlations with pulmonary function parameters and a very strong positive correlation with functional capacity, while strong negative correlations were observed with sleep quality and depression (*p* < 0.05). Smoking duration was strongly associated with poorer functional and psychosocial outcomes. **Conclusions:** Smoking is associated with early and multidimensional impairments in diaphragm muscle morphology, pulmonary function, functional capacity, and psychosocial status, even in individuals without overt respiratory disease. Reduced inspiratory diaphragm thickness may represent an early and clinically meaningful marker of smoking-related respiratory muscle dysfunction.

## 1. Introduction

The use of tobacco and tobacco products is not only the most significant independent cause of preventable deaths worldwide, but also one of the most serious and widespread public health issues. Every year, millions of people die from tobacco-related diseases and face conditions with high morbidity, such as cardiovascular, pulmonary, and malignant pathologies. It is reported that approximately 1 billion people currently use tobacco products. Although tobacco use prevalence is expected to decline over the next 10 years, the total number of smokers is expected to remain high due to global population growth [1]. Cigarette smoke contains over 7000 chemical substances (nitrogen, oxygen, carbon monoxide, water, argon, hydrogen, acetone, nitrogen oxides, sulfur compounds, etc.) that affect the phenotype and function of immune cells infiltrating the lungs [2,3,4]. Exposure of the lungs to these toxic pollutants causes oxidative stress in the epithelial cells of the alveoli, resulting in irreversible damage to the lung parenchyma [5,6,7]. The diaphragm is the primary respiratory muscle that increases lung volume during inspiration and provides effective ventilation. Long-term smoking is associated with atrophy, structural changes, and decreased contractile function in the diaphragm muscle fibers. This decrease in diaphragm muscle function may be an important risk factor that increases susceptibility to pneumonia and respiratory tract infections, particularly during the aging process [8]. Although the effects of smoking on the respiratory system have been extensively studied, there are limited studies on the structural and functional characteristics of the diaphragm muscle in healthy smokers [9,10]. Although relationships between diaphragm thickness and movement and lung function have been reported in COPD populations, there are only a limited number of studies examining these relationships in healthy smokers [11].

The aim of this study is to evaluate diaphragm function in individuals with cigarette addiction using ultrasonography and to investigate possible changes in respiratory capacity, thereby revealing the effects of smoking on respiratory muscles and contributing to clinical evaluation and rehabilitation approaches.

## 2. Methods

This prospective cross-sectional, and analytical observational clinical research was approved by the clinical research ethics committee of Inonu University (2023/5347). This study was conducted in the Physiotherapy and Rehabilitation Department of Faculty of Health Sciences at Inonu University in accordance with the Declaration of Helsinki. Participants who met the criteria and gave informed consent were included in the study. They signed the consent form.

Between January 2025 and April 2025, twenty participants who applied to the Smoking Cessation Clinic of Inonu University Turgut Ozal Medical Faculty Hospital, and twenty participants who had never smoked and had no history of long-term smoke exposure, were included in the study.

The inclusion criteria were being between 25 and 65 years of age and having a smoking history of at least 20 pack-years.

The exclusion criteria were being diagnosed with vestibular, neurological, or orthopedic disorders which may affect balance and mobility; uncontrolled hypertension; uncontrolled arrhythmia; chronic obstructive lung disease; rheumatic valvular heart disease; previous heart valve surgery; recent coronary bypass surgery (three months prior to study); acute myocardial infarction, inadequate cooperation; renal impairment; cognitive or mental impairments; pregnancy or recent plans for pregnancy and a history of substance abuse other than smoking.

All participants were evaluated for pulmonary function, walking capacity, lower extremity strength, sleep quality and depression level by a physiotherapist. Diaphragm muscle thickness during both inspiration and expiration was measured by a radiologist. The radiologist was blinded to group allocation.

### 2.1. Outcome Measures

The outcome measures used in this study were as follows:

#### 2.1.1. Pulmonary Function Test

Pulmonary functions were measured with spirometry (Cosmed Pony FX, Rome, Italy) according to the criteria of the American Thoracic Society and European Respiratory Society. The best value from three acceptable tests was recorded. Forced vital capacity (FVC), forced expiratory volume in 1 sec (FEV_1_), forced vital capacity/forced expiratory volume in 1-sec ratio (FEV_1_/FVC), and peak expiratory flow (PEF) were measured and expressed as the percentages of the predicted values [12,13].

#### 2.1.2. 6 min Walking Test

Functional capacity was determined by the 6 min walking test (6 MWT) according to the criteria of the American Thoracic Society. Participants were instructed to walk as fast as possible between two cones positioned 30 m apart and the distance walked in 6 min was recorded in meters [14].

#### 2.1.3. Sit-to-Stand Tests

The 30 s sit-to-stand test (30s-STST) is widely used to assess lower extremity muscle strength, functional capacity, and physical performance. It provides indirect information about the strength of the knee and hip extensor muscles, which are crucial for daily activities such as rising from a chair or climbing stairs. In addition, the test reflects balance, postural control, and muscular endurance [15]. A standard 45 cm chair without armrests was used for the test. Participants were asked to sit near the front edge of the chair with their feet flat on the floor and were instructed to stand up until their knees and hips were fully extended, and sit down with their arms crossed over the chest. They were then asked to repeat this sit-to-stand maneuver as quickly as possible for 30 s. At the end of the test, the total number of completed repetitions was recorded [16].

#### 2.1.4. Pittsburgh Sleep Quality Index

Pittsburgh sleep quality index (PSQI) was used to evaluate sleep quality. The PSQI is a self-report scale that evaluates sleep quality and disturbance over a one-month period. The scale consists of 24 questions, 19 of which are answered by the individual, and 5 are filled out by the individual’s bedmate. Questions answered by the individual are evaluated, while questions answered by the bedmate are not. The 19 questions answered by the individual evaluate 7 sub-dimensions, including subjective sleep quality, sleep latency, sleep duration, habitual sleep efficiency, sleep disturbance, use of sleeping pills, and daytime dysfunction. The sum of the seven component scores gives the total PSQI score. The total score ranges from 0 to 21, with a score above 5 indicating poor sleep quality [17,18].

#### 2.1.5. Beck Depression Inventory

The Beck Depression Inventory (BDI) is a questionnaire used to evaluate an individuals’ mental state. It consists of 21 questions that the individual answers according to their current state. Each question has 4 response options scored from 0 to 3. Total scores of 0–9 are interpreted as no depression or minimal depression, 10–18 as mild depression, 19–29 as moderate depression, and 30–63 as severe depression. The Turkish validity and reliability study was conducted [19,20].

#### 2.1.6. Diaphragm Muscle Ultrasound Assessment

The diaphragm muscle was assessed using B-mode ultrasonography with a high-frequency linear probe (7–12 MHz). All examinations were performed in the Department of Radiology, Inonu University Faculty of Medicine, by an experienced radiologist. Measurements were obtained from the right hemidiaphragm with participants in the supine position, along the mid-axillary line at the lower lateral hemithorax, specifically at the 8th–9th intercostal space corresponding to the zone of apposition of the diaphragm.

B-mode imaging was preferred because the primary outcome was diaphragm thickness, which requires clear visualization of the pleural and peritoneal diaphragmatic layers and is optimally assessed using high-frequency B-mode ultrasonography. Accordingly, the assessment focused exclusively on absolute diaphragm thickness measurements, and diaphragmatic thickening fraction was not calculated.

Diaphragm thickness was measured at end-expiration (functional residual capacity) and end-inspiration. Measurements obtained during inspiratory and expiratory phases were used for comparative analysis between smokers and healthy non-smoking individuals under standardized conditions.

For each condition, at least three consecutive measurements were recorded, and the mean value was included in the analysis. Device calibration and measurement standardization were performed prior to data collection to ensure reliability.

Limits of normality were not established, as the study was designed for group-wise comparative analysis rather than defining diagnostic reference values, which vary with demographic and methodological factors. This standardized B-mode approach has been reported as reliable and reproducible for diaphragm thickness assessment at the zone of apposition [21,22].

#### 2.1.7. Statistical Analysis

Sample size was calculated using G*Power 3.1 software. Based on calculations using Cohen’s d = 1.0, α = 0.05, and power = 0.80 parameters for diaphragm muscle thickness, it was determined that at least 17 participants per group would be sufficient. To increase statistical power, 20 individuals were included in each group. In the analysis of the data, independent samples *t*-test or Mann–Whitney U test, Pearson or Spearman correlation analyses, and non-parametric tests when necessary were used. All analyses were performed using SPSS v25, and the significance level was set at *p* < 0.05. The raw data underlying the statistical analyses are available in Supplementary File S1.

## 3. Results

Thirty-five smokers were assessed for eligibility. Ten individuals were excluded for not meeting the inclusion criteria, and five declined to participate. Twenty-five age-matched never-smokers were assessed for the control group; five individuals were excluded due to refusal to participate. A total of 20 participants were included in each group. Among the smokers, six individuals had hypertension and three had diabetes mellitus. Comparison of the demographic characteristics between the groups revealed that body mass index (BMI) was significantly higher in the smoking group.

The demographic and clinical characteristics of the participants are presented in Table 1, while the assessments of pulmonary function, functional capacity, lower extremity strength, sleep quality, depression level and diaphragm muscle thickness are shown in Table 2.

Significant differences were observed between the groups in all assessed parameters except for expiratory diaphragm thickness (*p* < 0.05). Effect size analysis demonstrated large to very large effects for FEV_1_ and FEF_25–75_% parameters, functional capacity, and inspiratory diaphragm thickness *(r*: 0.78–0.86) (Table 2).

The relationships between inspiratory–expiratory diaphragm muscle thickness and pulmonary function, walking capacity, lower extremity muscle strength, sleep quality, and depression level in smokers are summarized in Table 3.

In smokers, analysis of the relationships between inspiratory diaphragm thickness and other parameters revealed moderate to strong positive correlations with all pulmonary function test parameters (*r* = 0.45–0.66; *p* < 0.05). A very strong positive correlation was observed between inspiratory diaphragm thickness and functional capacity (*r* = 0.726; *p* < 0.05). In contrast, strong negative correlations were found with sleep quality and depression level (*r* = −0.67 to −0.68; *p* < 0.05). No significant correlations were observed between expiratory diaphragm thickness and any of the examined parameters (*p* > 0.05) (Table 3).

The relationships between smoking duration and inspiratory–expiratory diaphragm muscle thickness, pulmonary function, walking capacity, lower extremity muscle strength, sleep quality, and depression level are presented in Table 4.

In smokers, smoking duration was moderately negatively correlated with FEF_25–75_%. Smoking duration showed very strong negative correlations with the 6-MWT distance (*r* = −0.849; *p* < 0.05), the 30 s-STST performance *(r* = −0.703; *p* < 0.05) and inspiratory diaphragm muscle thickness *(r* = −0.735; *p* < 0.05). In contrast, smoking duration was very strongly positively correlated with the total PSQI score (*r* = 0.823; *p* < 0.05) and moderately to strongly positively correlated with the BDI score (*r* = 0.592; *p* = 0.05). No significant correlations were observed between smoking duration and expiratory diaphragm thickness, FVC%, FEV_1_%, FEV_1_/FVC, PEF% (*p* > 0.05) (Table 4).

## 4. Discussion

This study demonstrates that cigarette addiction has multidimensional and interrelated adverse effects on diaphragm function, respiratory capacity, functional capacity, sleep quality, and emotional state.

In our study, the significantly lower FVC, FEV_1_, FEV_1_/FVC ratio, PEF, and especially FEF_25–75_ values in smokers indicate that smoking has adverse effects on respiratory functions. The literature reports that cigarette smoke causes inflammation, epithelial damage, and impaired mucociliary activity in the airways, resulting in functional loss in both the central and peripheral airways [23,24,25]. Previous studies have found that cigarette smoke causes inflammation, fibrosis, and premature closure in the small airways, resulting in a significant decrease in FEF_25–75_% values [25]. Our study results from pulmonary function tests show that both small and large airways are affected in smokers, consistent with the literature. Although FEF_25–75_% has been proposed as a marker of small airway dysfunction, its clinical utility for early detection remains controversial. The ERS/ATS guidelines emphasize that evidence supporting the use of small airway indices as standalone diagnostic tools is insufficient [26]. In our study, the significant correlations observed between FEF_25–75_% values and inspiratory diaphragm thickness as well as functional capacity suggest that this parameter may be associated with pathophysiological changes reflecting small airway dysfunction. However, these findings indicate that FEF_25–75_% should be interpreted within a multidimensional assessment framework rather than being considered a standalone tool for early diagnosis. In the literature, alternative modalities such as impulse oscillometry (IOS), multiple-breath nitrogen washout (MBNW), and advanced imaging techniques have been reported to be more sensitive for the assessment of small airway function, particularly for detecting early pathological changes. Because these methods are largely effort-independent, they may identify small airway dysfunction at earlier stages compared with conventional spirometry. IOS provides information on peripheral airway resistance and reactance, while MBNW allows detailed evaluation of ventilation heterogeneity, both of which are considered sensitive markers of early small airway involvement [27,28]. In future studies, the combined use of diaphragm ultrasonography with these advanced modalities may contribute to a more comprehensive assessment of early smoking-related respiratory system alterations.

Our study demonstrates that the thickness of the diaphragm muscle during the inspiration phase is significantly reduced in individuals who smoke, indicating that smoking has structural and functional effects on the respiratory muscles. The literature reports that chronic exposure to tobacco smoke increases oxidative stress levels, activating proteolytic pathways, and that these mechanisms may pave the way for the development of atrophy in the respiratory muscles [3,9]. Impaired mitochondrial morphology and loss of muscle homeostasis are among the possible pathophysiological mechanisms that could explain the decrease in diaphragm contractility associated with smoking [9]. However, the detection of structural abnormalities in the diaphragm fiber architecture in smokers without any clinical disease suggests that morphological changes in the diaphragm may begin before the development of disease and that smoking may have early effects on muscle tissue [8].

The positive relationship between diaphragm thickness during inspiration and respiratory function parameters such as FEV_1_, FVC, PEF, and FEF_25–75_% supports the notion that diaphragm function is one of the key determinants of ventilatory capacity [3,8,9,10]. From a physiological perspective, diaphragm contraction occurs predominantly during the inspiratory phase, when the muscle actively shortens to generate negative intrathoracic pressure, whereas expiration at rest is largely passive and mainly driven by the elastic recoil of the lung–chest wall system. In this context, Carrillo-Esper et al. demonstrated that standardized ultrasonographic measurements of diaphragm thickness performed during inspiration in the zone of apposition provide more reproducible and clinically meaningful data [29]. Accordingly, inspiratory ultrasonographic assessment more accurately reflects diaphragm muscle activation, loading, and functional adaptation, while expiratory measurements mainly represent passive structural characteristics and are less sensitive to functional impairment. Consistent with this view, Spiesshoefer et al. reported that inspiratory diaphragm thickness and thickening-related indices show stronger associations with respiratory muscle strength and clinical outcomes, whereas expiratory measurements are less informative for detecting early dysfunction [9,10,30]. Therefore, the absence of significant differences in expiratory diaphragm thickness observed in our study may be considered a physiologically expected finding and suggests that smoking-related diaphragm involvement is primarily mediated through increased inspiratory muscle loading and elevated ventilatory demand, consistent with the existing literature [8,10].

Functional capacity assessments reveal that smokers’ performance on the 6-min Walk Test and the 30 s sit-to-stand test is significantly lower, highlighting the systemic effects of smoking. The literature shows that exercise tolerance is reduced in smokers due to decreased oxygen transport capacity, impaired muscle perfusion, and early onset of muscle fatigue [6,9,10]. In our study, the strong positive correlation between inspiratory diaphragm thickness and functional capacity tests is consistent with the literature and supports the decisive role of respiratory muscle function in exercise performance [6,10,23]. Furthermore, the strong negative correlations found between smoking duration and functional capacity suggest that the cumulative effects of smoking may increase physical performance loss.

In our study, the high PSQI scores found in smokers are consistent with the literature on nicotine’s stimulatory effects on the central nervous system and its disruptive role in sleep architecture (increased sleep latency, increased nighttime awakenings, and impaired daytime functioning) [31]. Similarly, the high depression scores among smokers in our study support the bidirectional relationship between smoking and depressive symptoms [32]. In our study, inspiratory diaphragm thickness was negatively correlated with PSQI and BDI scores, indicating that reduced diaphragm function is associated with poorer sleep quality and a higher emotional symptom burden. However, these findings should not be interpreted as evidence of a direct causal relationship. Rather, they may reflect a shared clinical and physiological phenotype in which respiratory muscle dysfunction, increased ventilatory load, dyspnea perception, sleep disturbance, and emotional distress coexist. Previous studies in chronic respiratory populations have reported associations between respiratory muscle impairment, sleep disruption, and psychological symptoms. For example, patients with asthma and COPD frequently exhibit poor sleep quality alongside higher rates of anxiety and depression, consistent with a broader disease phenotype rather than isolated dysfunction [33]. In COPD specifically, increasing dyspnea severity has been linked to deteriorating sleep quality [34], and comorbid sleep disturbances with depressive and anxiety symptoms have been shown to cluster together in patients with chronic airflow limitation [35]. Additionally, studies in smokers and substance use populations highlight the co-occurrence of respiratory muscle weakness, dyspnea, and sleep quality impairment [36].

The significant relationships found between smoking duration and inspiratory diaphragm thickness, functional capacity, sleep quality, and depression level reveal the increasing and multidimensional effects of smoking addiction over time. These findings support the notion that the assessment process for smokers should not be limited to respiratory function tests alone; rather, it should be approached holistically, incorporating diaphragm function, functional capacity, and psychosocial variables.

A notable finding in our study is that BMI was significantly higher in the smoking group compared to controls, a condition that would typically be expected to increase diaphragm thickness due to elevated abdominal loading [37]. However, despite this potential mechanical advantage, inspiratory diaphragm thickness was significantly reduced in smokers. This paradoxical finding may reflect early sarcopenia-like changes in the diaphragm muscle induced by chronic cigarette exposure. Smoking-related oxidative stress and inflammation are known to activate proteolytic pathways, impair mitochondrial function, and reduce muscle fiber integrity, leading not only to a reduction in muscle mass but also to deterioration in muscle quality [3,9]. Therefore, the observed reduction in inspiratory diaphragm thickness in smokers with higher BMI may indicate impaired muscle quality rather than insufficient mechanical loading. In line with this interpretation, Shinohara et al. demonstrated that diaphragm dysfunction assessed by ultrasonography is closely associated with sarcopenia-related features, emphasizing the importance of evaluating not only muscle thickness but also functional parameters such as thickening fraction and contractility [38]. Future studies incorporating comprehensive ultrasound-based assessments and controlling for BMI using ANCOVA may help clarify the interaction between smoking, body composition, and diaphragm sarcopenic changes.

## 5. Limitations

This study has several limitations that should be acknowledged. First, the cross-sectional design does not allow causal inferences regarding the relationship between smoking exposure and diaphragm muscle alterations. Second, the relatively small sample size may limit the generalizability of the findings and reduce statistical power for subgroup analyses. Third, although body mass index was significantly higher in the smoking group and may influence diaphragm morphology through increased abdominal loading, this potential confounding effect was not statistically controlled. Future studies incorporating body composition analysis and covariate-adjusted models may provide clearer insight into the independent effects of smoking on diaphragm structure and function. In addition, diaphragm ultrasonography is an operator-dependent technique, and although measurements were performed by an experienced radiologist using a standardized protocol, subtle inter- and intra-observer variability cannot be fully excluded. Finally, the study population consisted of individuals without clinically diagnosed respiratory disease; therefore, the findings may not be directly extrapolated to patients with established pulmonary pathology.

### Future Directions and Clinical Implications

Future research should focus on longitudinal designs to determine whether changes in inspiratory diaphragm thickness precede the development of clinically overt respiratory disease and to evaluate the effects of smoking cessation on diaphragm morphology and function. Incorporating additional ultrasonographic indices such as diaphragm excursion, thickening fraction, and muscle quality parameters, as well as effort-independent assessments of small airway function, may further enhance the understanding of early smoking-related respiratory alterations.

From a clinical perspective, diaphragm ultrasonography represents a non-invasive, accessible, and radiation-free assessment tool that may complement conventional pulmonary function testing in smokers. The identification of reduced inspiratory diaphragm thickness in individuals without overt respiratory disease suggests a potential role for ultrasonography in early risk stratification and monitoring. Moreover, these findings highlight the importance of integrating respiratory muscle assessment into preventive and rehabilitative strategies, including targeted physiotherapy and early intervention programs within smoking cessation settings.

## 6. Conclusions

This study demonstrates that cigarette smoking is associated with early impairments in inspiratory diaphragm muscle thickness, pulmonary function, functional capacity, and psychosocial status, even in individuals without clinically diagnosed respiratory disease. Among the evaluated parameters, reduced inspiratory diaphragm thickness showed strong associations with respiratory function, functional performance, and smoking exposure, suggesting that diaphragm ultrasonography may provide clinically meaningful information beyond conventional spirometry. These findings support the potential role of inspiratory diaphragm thickness as an early marker of smoking-related respiratory muscle dysfunction.

## Figures and Tables

**Table 1:** Demographic and clinic characteristics of smoking and non-smoking groups.

	Control Group (*n* = 20)	Smoking Group (*n* = 20)	*p* Value
Age (years)	44.40 ± 7.19	45.90 ± 7.01	0.508
Gender (Female/Male)	11/9	10/10	1.000
Height (cm)	170.15 ± 7.43	166.75 ± 8.22	0.178
Weight (kg)	70.15 ± 14.82	77.10 ± 16.76	0.173
Body mass index (kg/m^2^)	24.11 ± 4.25	27.85 ± 6.25	**0.033** *
Education (years)	High school; %60 University; %40	High school; %40 University; %60	0.527
Number of pack-years	-	34.25 ± 5.84	-
Dyspnea level (mMRC)	0.35 ± 0.48	2.20 ± 0.60	**<0.001** *

* Data are reported as mean ± standard deviation, median (min–max) and *n* (%). **Control group:** Non-smoking group. **Abbreviations:** mMRC, Modified Medical Research Council Scale.

**Table 2:** Comparison of the pulmonary function, functional capacity, lower extremity muscle strength, sleep quality, depression level, and inspiratory–expiratory diaphragm muscle thickness between groups.

	Control Group (*n* = 20)	Smoking Group (*n* = 20)	*p* Value	ES (*r*)
FVC%	91.89 ± 9.08	81.40 ± 11.36	0.029	−0.351
FEV_1_%	95.79 ± 6.48	73.09 ± 15.86	<0.001	−0.784
FEV_1_/FVC(%)	89.21 ± 7.86	74.58 ± 11.66	<0.001	−0.666
PEF%	86.92 ± 7.12	69.51 ± 10.04	<0.001	−0.675
FEF_25–75_%	82.71 ± 6.27	61.65 ± 11.19	<0.001	−0.781
6 WMT (m)	597.85 ± 16.94	405.80 ± 24.65	<0.001	−0.867
30s-STST (repetitions)	12.80 ± 0.89	9.65 ± 0.67	<0.001	−0.878
Total PSQI score	2.00 ± 0.65	6.70 ± 1.08	<0.001	+0.883
Mean BDI score	5.40 ± 1.35	9.75 ± 2.10	<0.001	+0.812
Inspiratory thickness (mm)	5.90 ± 0.21	5.04 ± 0.21	<0.001	−0.866
Expiratory thickness (mm)	3.09 ± 0.15	3.02 ± 0.14	0.145	−0.236

Data are presented as mean ± SD. **Note:** Normally distributed data analyzed with independent *t*-test, Non-normally distributed data analyzed with Mann–Whitney U test. **Abbreviations:** FVC, forced vital capacity; FEV_1_, forced expiratory volume in 1 s; FEF_25–75_, forced mid-expiratory flow between 25% and 75%; PEF, peak expiratory flow; 6 MWT, 6 min walking test; 30s-STST 30 s STS sit-to-stand test; PSQI, Pittsburgh Sleep Quality Index; BDI, Beck Depression Inventory.

**Table 3:** Correlations between pulmonary function, functional capacity, lower extremity muscle strength, sleep quality, depression level, and inspiratory–expiratory diaphragm muscle thickness in smokers.

	Inspiratory Thickness (mm)		Expiratory Thickness (mm)	
	*r*	*p* Value	*r*	*p* Value
FVC%	0.447	0.048	0.350	0.131
FEV_1_%	0.466	0.038	0.318	0.171
FEV_1_/FVC(%)	0.473	0.035	−0.084	0.724
PEF%	0.560	0.011	0.228	0.333
FEF_25–75_%	0.563	0.011	0.278	0.235
6 WMT (m)	0.726	<0.001	0.244	0.304
30s-STST (repetitions)	0.477	0.033	0.161	0.497
Total PSQI score	−0.671	0.001	−0.139	0.559
Mean BDI score	−0.688	0.001	−0.171	0.470

Abbreviations: FVC, forced vital capacity; FEV_1_, forced expiratory volume in 1 s; FEF_25–75_, forced mid-expiratory flow between 25% and 75%; PEF, peak expiratory flow; 6 MWT, 6 min walking test; 30s-STST, 30 s STS sit-to-stand test; PSQI, Pittsburgh Sleep Quality Index; BDI, Beck Depression Inventory.

**Table 4:** Correlations between pulmonary function, functional capacity, lower extremity muscle strength, sleep quality, depression level, and smoking period in smokers.

	Smoking Period (Years)	
	*r*	*p* Value
FEF_25–75_%	−0.465	0.039
6 WMT (m)	−0.849	<0.001
30s-STST (repetitions)	−0.703	<0.001
Total PSQI score	+0.823	<0.001
Mean BDI score	+0.592	0.006
Inspiratory thickness (mm)	−0.735	<0.001

Abbreviations: FEF_25–75_, forced mid-expiratory flow between 25% and 75%; PEF, peak expiratory flow; 6 MWT, 6-min walking test; 30s-STST, 30-s STS sit-to-stand test; PSQI, Pittsburgh Sleep Quality Index; BDI, Beck Depression Inventory.

## Data Availability

The data presented in this study are not publicly available due to ethical and privacy considerations but are available from the corresponding author upon reasonable request.

## Supplementary Materials

The following supporting information can be downloaded at https://www.mdpi.com/article/10.3390/jcm15051950/s1, Supplementary File S1: The raw data underlying the statistical analyses.
